# Supplementary figures and images for: Insights on cross-species transmission of SARS-CoV-2 from structural modeling
Source: PLoS Comput Biol. 2020 Dec 3;16(12):e1008449. doi: 10.1371/journal.pcbi.1008449 (PMC7714162; doi:10.1371/journal.pcbi.1008449)

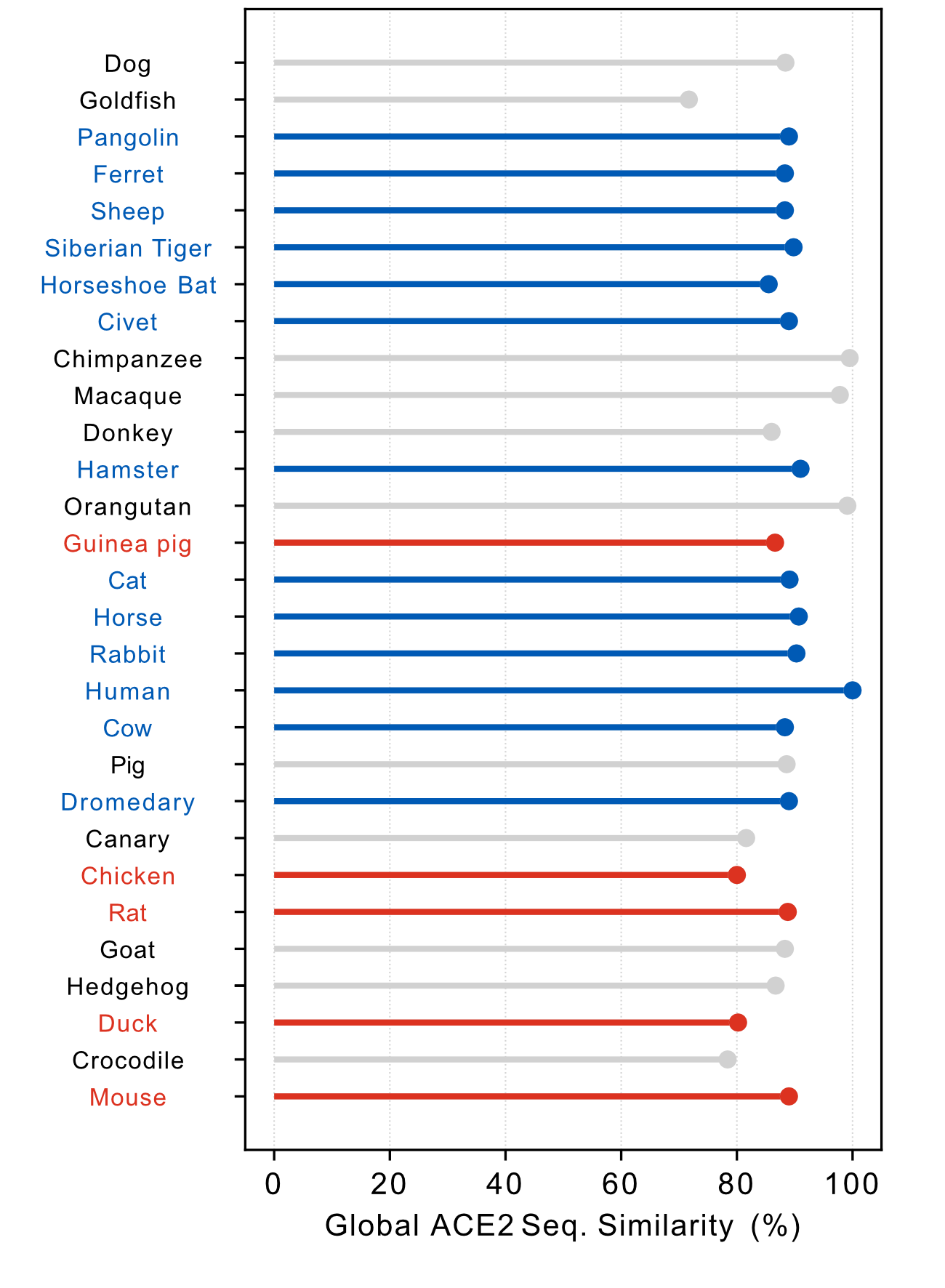

Supplement: S1 Fig — (TIFF) [file pcbi.1008449.s001.tiff]

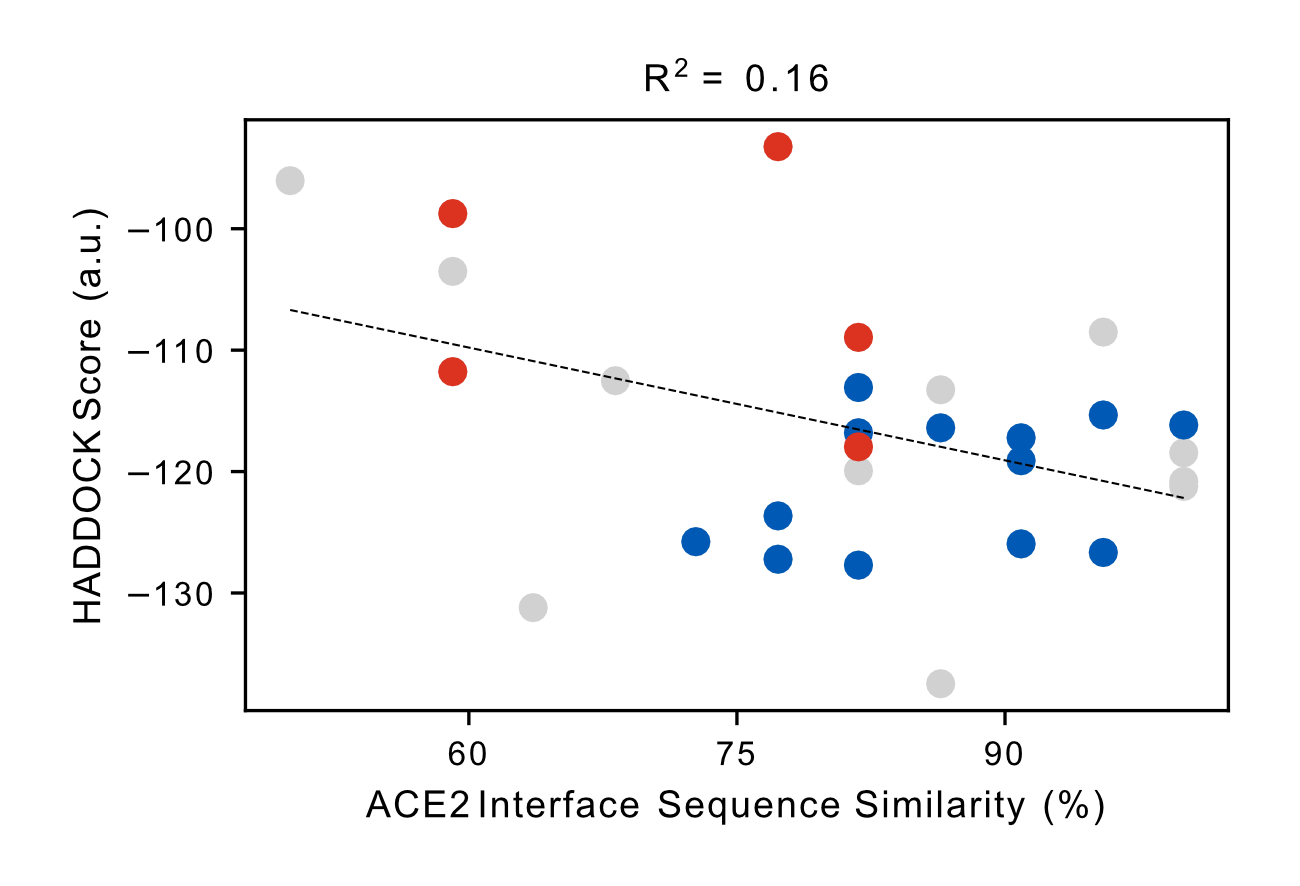

Supplement: S2 Fig — (TIFF) [file pcbi.1008449.s002.tiff]

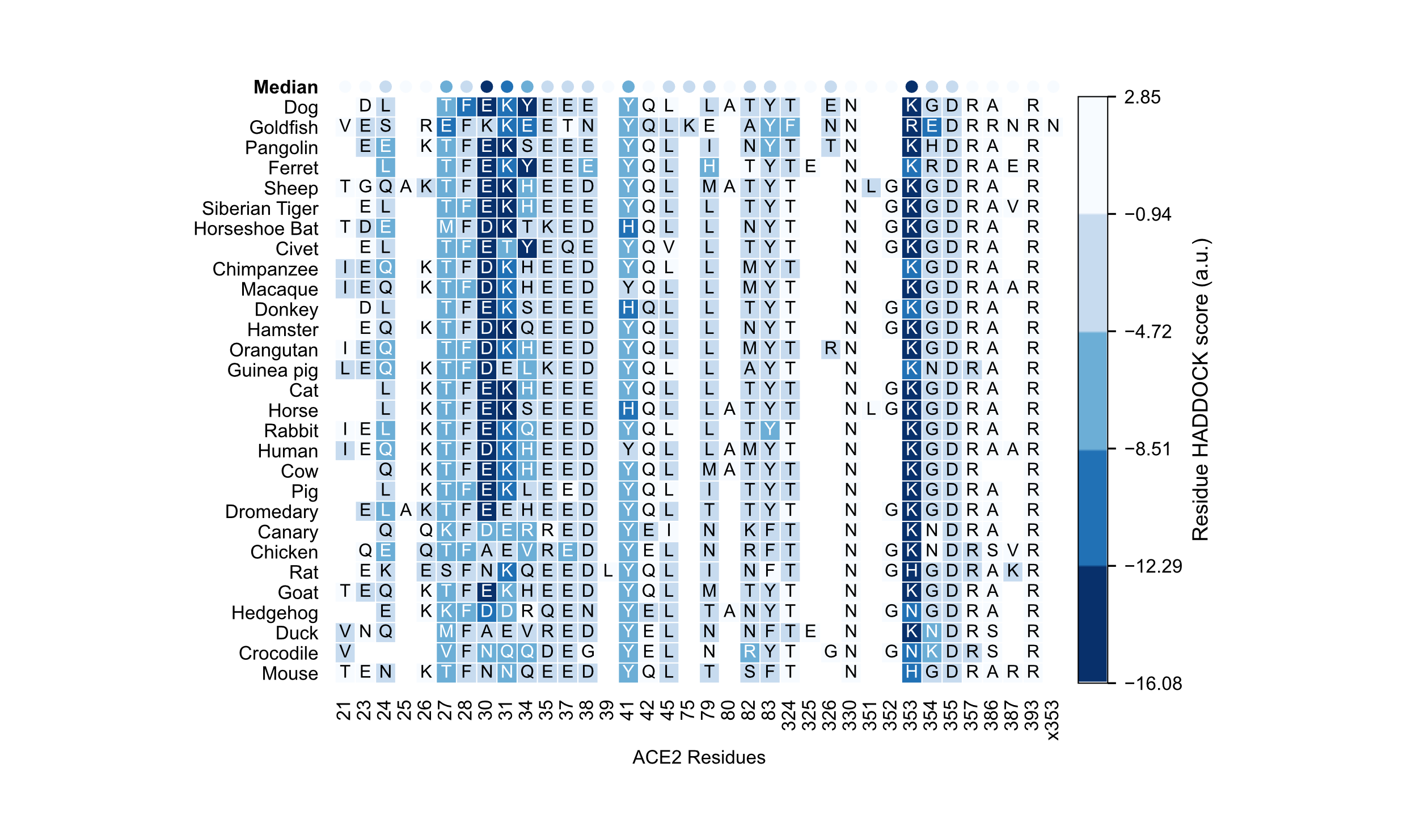

Supplement: S3 Fig — (TIFF) [file pcbi.1008449.s003.tiff]

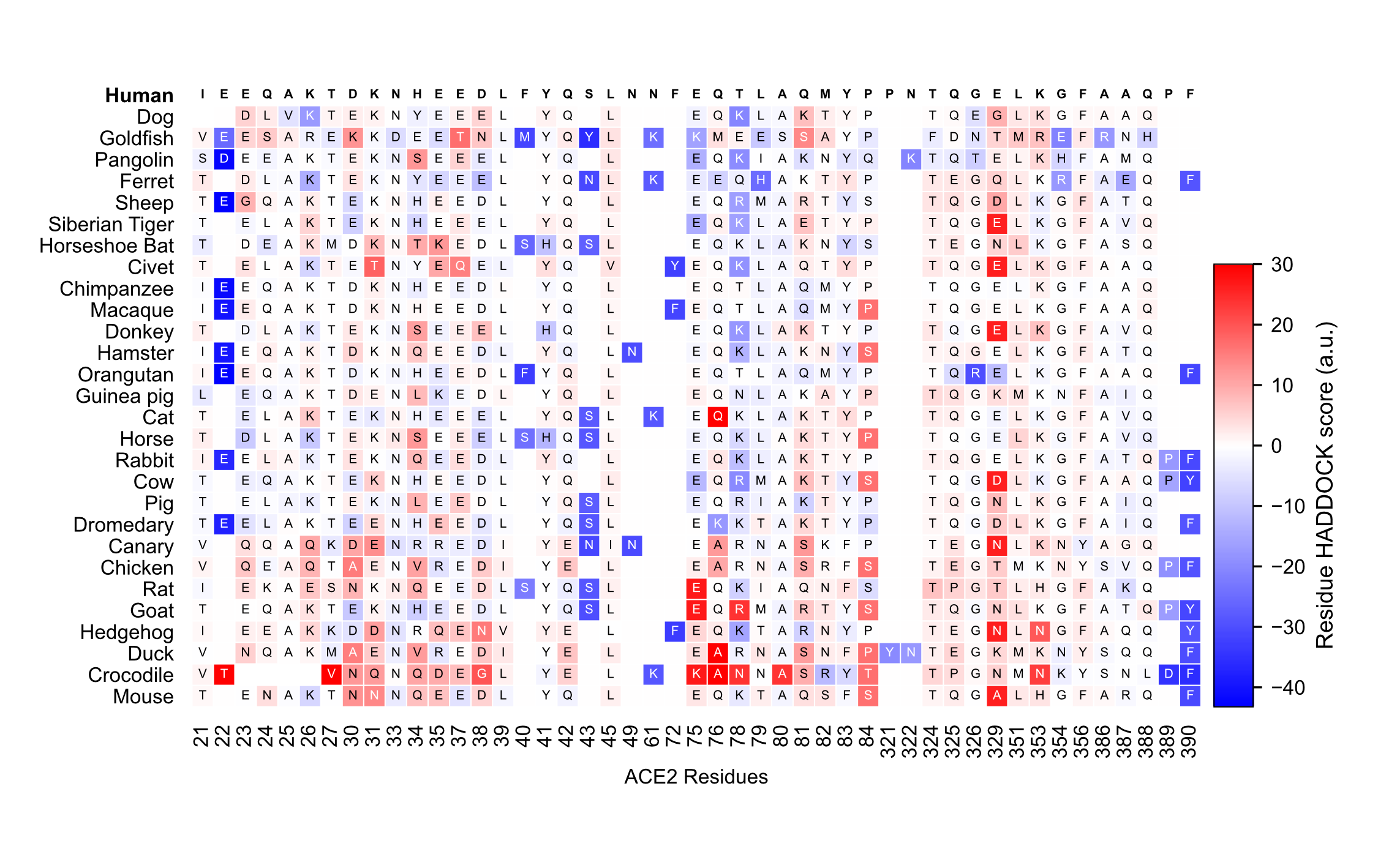

Supplement: S4 Fig — (TIFF) [file pcbi.1008449.s004.tiff]
