# Supplementary material for: Insights on cross-species transmission of SARS-CoV-2 from structural modeling
Source: PLoS Comput Biol. 2020 Dec 3;16(12):e1008449. doi: 10.1371/journal.pcbi.1008449 (PMC7714162; doi:10.1371/journal.pcbi.1008449)
Supplement: S1 Table — (DOCX) [file pcbi.1008449.s005.docx]

**Table S1. Interface contacts of refined human ACE2:RBD**

| **Type** | **ACE2 Residue** | **RBD Residue** | **Frequency in Top 10 hACE2 Models** | **In crystal (6m0j)** |
| --- | --- | --- | --- | --- |
| hbond | Q24 | A475 | 7 |  |
| hbond | Q24 | N487 | 6 | **x** |
| hbond | T27 | Y489 | 9 |  |
| ionic | D30 | K417 | 10 | **x** |
| hbond | K31 | F490 | 4 |  |
| hbond | K31 | Q493 | 10 | **x** |
| hbond | H34 | Y453 | 2 |  |
| hbond | E35 | Q493 | 6 | **x** |
| hbond | E37 | Y505 | 4 | **x** |
| hbond | D38 | Y449 | 10 | **x** |
| hbond | Y41 | T500 | 10 | **x** |
| hbond | Y83 | N487 | 2 | **x** |
| hbond | K353 | Y495 | 1 |  |
| hbond | K353 | G496 | 9 |  |
| hbond | K353 | G502 | 10 | **x** |
