# Supplementary material for: Insights on cross-species transmission of SARS-CoV-2 from structural modeling
Source: PLoS Comput Biol. 2020 Dec 3;16(12):e1008449. doi: 10.1371/journal.pcbi.1008449 (PMC7714162; doi:10.1371/journal.pcbi.1008449)
Supplement: S2 Table — The values represent the average and standard deviation of the 10 best models (ranked by HADDOCK score) of each species. (DOCX) [file pcbi.1008449.s006.docx]

**Table S2.** **HADDOCK scores and individual energy terms for each modeled ACE2:RBD complex.** The values represent the average and standard deviation of the 10 best models (ranked by HADDOCK score) of each species.

| **Species** | **HADDOCK Score**  **(a.u.)** | **van der Waals**  **(kcal/mol)** | **Electrostatics**  **(kcal/mol)** | **Desolvation**  **(a.u.)** | **Buried Surface Area**  **(Å^2^)** |
| --- | --- | --- | --- | --- | --- |
| Dog | -137,5 ± 3,7 | -64,5 ± 4,0 | -230,3 ± 18,4 | -26,9 ± 2,5 | 1903 ± 44 |
| Ferret | -127,2 ± 1,8 | -60,6 ± 2,6 | -195,8 ± 16,8 | -27,5 ± 3,4 | 1824 ± 70 |
| Goldfish | -131,2 ± 5,9 | -68,2 ± 3,8 | -189,3 ± 19,2 | -25,1 ± 3,2 | 1925 ± 67 |
| Pangolin | -127,7 ± 3,3 | -59,9 ± 3,6 | -233,8 ± 16,0 | -21,0 ± 2,0 | 1854 ± 32 |
| Hamster | -119,1 ± 3,1 | -57,6 ± 2,8 | -242,8 ± 23,8 | -13,0 ± 2,5 | 1821 ± 51 |
| Siberian Tiger | -126,0 ± 4,8 | -60,7 ± 3,9 | -196,3 ± 27,8 | -26,0 ± 3,3 | 1804 ± 37 |
| Guinea pig | -118,0 ± 3,3 | -62,3 ± 2,6 | -163,3 ± 12,2 | -23,0 ± 2,5 | 1868 ± 41 |
| Sheep | -126,7 ± 2,5 | -61,4 ± 2,2 | -231,2 ± 26,8 | -19,0 ± 3,6 | 1876 ± 25 |
| Chimpanzee | -121,2 ± 3,4 | -57,5 ± 3,7 | -213,5 ± 17,6 | -21,0 ± 3,1 | 1816 ± 33 |
| Civet | -123,6 ± 4,2 | -56,4 ± 4,5 | -193,6 ± 27,9 | -28,6 ± 3,4 | 1786 ± 56 |
| **Human** | -116,2 ± 3,2 | -54,4 ± 2,8 | -221,8 ± 15,1 | -17,5 ± 3,2 | 1781 ± 37 |
| Dromedary | -113,1 ± 2,2 | -56,0 ± 2,0 | -181,3 ± 16,3 | -20,8 ± 2,7 | 1733 ± 65 |
| Horseshoe bat | -125,8 ± 2,7 | -60,7 ± 3,2 | -259,4 ± 17,5 | -13,2 ± 3,2 | 1819 ± 52 |
| Pig | -113,3 ± 1,1 | -53,8 ± 2,1 | -202,7 ± 12,6 | -18,9 ± 2,5 | 1715 ± 32 |
| Cow | -115,3 ± 4,1 | -54,9 ± 1,5 | -212,1 ± 19,1 | -18,0 ± 4,0 | 1766 ± 41 |
| Cat | -117,2 ± 1,7 | -55,2 ± 2,0 | -202,4 ± 9,8 | -21,5 ± 3,0 | 1752 ± 42 |
| Macaque | -120,8 ± 3,2 | -56,5 ± 2,1 | -217,7 ± 18,6 | -20,7 ± 2,3 | 1841 ± 37 |
| Orangutan | -118,5 ± 4,5 | -62,5 ± 2,7 | -182,3 ± 21,1 | -19,5 ± 3,8 | 1837 ± 49 |
| Horse | -116,8 ± 4,2 | -55,4 ± 3,7 | -220,0 ± 14,0 | -17,4 ± 2,4 | 1762 ± 34 |
| Rabbit | -116,4 ± 5,6 | -55,6 ± 2,7 | -222,6 ± 18,2 | -16,2 ± 2,7 | 1827 ± 55 |
| Donkey | -119,9 ± 1,9 | -58,3 ± 1,7 | -220,5 ± 13,5 | -17,6 ± 3,3 | 1767 ± 44 |
| Goat | -108,5 ± 4,0 | -53,5 ± 3,6 | -189,0 ± 10,5 | -17,2 ± 3,1 | 1745 ± 64 |
| Chicken | -111,8 ± 1,4 | -64,8 ± 1,6 | -132,3 ± 15,0 | -20,5 ± 3,7 | 1802 ± 44 |
| Canary | -112,5 ± 2,8 | -61,5 ± 5,0 | -170,7 ± 33,7 | -16,8 ± 3,8 | 1822 ± 73 |
| Hedgehog | -103,5 ± 3,2 | -57,3 ± 5,3 | -160,9 ± 38,9 | -14,0 ± 3,8 | 1726 ± 48 |
| Rat | -108,9 ± 3,5 | -55,3 ± 4,5 | -162,4 ± 16,0 | -21,2 ± 2,7 | 1777 ± 58 |
| Crocodile | -96,0 ± 2,5 | -64,9 ± 2,7 | -90,4 ± 18,1 | -13,1 ± 2,6 | 1690 ± 49 |
| Duck | -98,8 ± 2,3 | -63,3 ± 1,9 | -99,5 ± 6,0 | -15,5 ± 3,1 | 1782 ± 55 |
| Mouse | -93,2 ± 2,6 | -53,8 ± 2,6 | -93,1 ± 14,4 | -20,8 ± 2,5 | 1598 ± 65 |
