# Supplementary material for: Insights on cross-species transmission of SARS-CoV-2 from structural modeling
Source: PLoS Comput Biol. 2020 Dec 3;16(12):e1008449. doi: 10.1371/journal.pcbi.1008449 (PMC7714162; doi:10.1371/journal.pcbi.1008449)
Supplement: S3 Table — The values represent the average and standard deviation of the 10 best models (ranked by HADDOCK score) of each model. (DOCX) [file pcbi.1008449.s007.docx]

**Table S3.** **HADDOCK scores and individual energy terms for mutated human ACE2:RBD and mouse ACE2:RBD complexes.** The values represent the average and standard deviation of the 10 best models (ranked by HADDOCK score) of each model.

| **Species** | **Variant** | **HADDOCK Score**  **(a.u.)** | **van der Waals**  **(kcal/mol)** | **Electrostatics**  **(kcal/mol)** | **Desolvation**  **(a.u.)** | **Buried Surface Area**  **(Å^2^)** |
| --- | --- | --- | --- | --- | --- | --- |
| **Human** | wild-type | -116,2 ± 3,2 | -54,4 ± 2,8 | -221,8 ± 15,1 | -17,5 ± 3,2 | 1781 ± 37 |
|  | D30A | -106 ± 2.7 | -56 ± 2.7 | -147 ± 11.6 | -20.6 ± 2.5 | 1764 ± 73 |
|  | D30N | -108.9 ± 2.8 | -58.3 ± 2.6 | -144.4 ± 17.1 | -21.7 ± 2.5 | 1790 ± 35 |
|  | K31E/E35R | -113.6 ± 3.5 | -58.2 ± 2.8 | -176.2 ± 18.4 | -20.1 ± 3.2 | 1788 ± 40 |
|  | K353H | -123 ± 4.7 | -56 ± 2.5 | -216.6 ± 23 | -23.7 ± 3.6 | 1782 ± 37 |
| **Mouse** | wild-type | -93,2 ± 2,6 | -53,8 ± 2,6 | -93,1 ± 14,4 | -20,8 ± 2,5 | 1598 ± 65 |
|  | N30D | -105.9 ± 1.9 | -54.8 ± 1.9 | -153.9 ± 7.6 | -20.3 ± 2.6 | 1607 ± 49 |
|  | H353K | -92.1 ± 3.4 | -55.1 ± 3.3 | -119.5 ± 15.7 | -13.1 ± 2.4 | 1629 ± 52 |
|  | N30D/N31K/H353K | -113.7 ± 2.4 | -56.5 ± 2.7 | -221.5 ± 24.9 | -12.9 ± 3.9 | 1816 ± 67 |
