# Supplementary material for: Insights on cross-species transmission of SARS-CoV-2 from structural modeling
Source: PLoS Comput Biol. 2020 Dec 3;16(12):e1008449. doi: 10.1371/journal.pcbi.1008449 (PMC7714162; doi:10.1371/journal.pcbi.1008449)
Supplement: S4 Table — The values represent the average and standard deviation of the 10 best models (ranked by HADDOCK score) of each model. (DOCX) [file pcbi.1008449.s008.docx]

**Table S4.** **HADDOCK scores and individual energy terms for mouse ACE2 bound to select frequent single-point mutants of RBD (in >0.01% of sequences) derived from an analysis of 104,979 SARS-CoV-2 genomes.** The values represent the average and standard deviation of the 10 best models (ranked by HADDOCK score) of each model.

| **Variant** | **Frequency** | **HADDOCK Score**  **(a.u.)** | **van der Waals**  **(kcal/mol)** | **Electrostatics**  **(kcal/mol)** | **Desolvation**  **(a.u.)** | **Buried Surface Area**  **(Å^2^)** |
| --- | --- | --- | --- | --- | --- | --- |
| wild-type | - | -93.2 ± 2.6 | -53.8 ± 2.6 | -93.1 ± 14.4 | -20.8 ± 2.5 | 1598 ± 65 |
| N439K | 265 (0.30%) | -97.2 ± 4.2 | -54.2 ± 2.5 | -110 ± 11.4 | -21 ± 2.1 | 1617 ± 38 |
| G446V | 15 (0.02%) | -96.7 ± 3.7 | -55.9 ± 3.3 | -99.5 ± 19.7 | -20.9 ± 2.7 | 1635 ± 63 |
| Y453F | 41 (0.05%) | -101.2 ± 3.3 | -56.9 ± 3.1 | -117.9 ± 14.1 | -20.7 ± 3.5 | 1616 ± 28 |
| L455F | 10 (0.01%) | -102.1 ± 2.6 | -54.5 ± 1.5 | -109.2 ± 14.1 | -25.7 ± 2.1 | 1646 ± 40 |
| A475V | 12 (0.01% | -98.8 ± 3.7 | -55.7 ± 2.6 | -112.7 ± 12.1 | -20.6 ± 2.4 | 1639 ± 36 |
| G476S | 18 (0.02%) | -98.2 ± 4.3 | -54.7 ± 2.3 | -126.6 ± 18.4 | -18.1 ± 3.2 | 1623 ± 68 |
| S477N | 3930 (4.44%) | -99.4 ± 5 | -56.8 ± 3.2 | -111 ± 20.1 | -20.4 ± 2.8 | 1612 ± 58 |
| T478I | 101 (0.11%) | -93.8 ± 3.1 | -53.7 ± 3.4 | -95.9 ± 13.6 | -20.9 ± 2.1 | 1609 ± 56 |
| P479S | 73 (0.08%) | -95 ± 1.9 | -54.3 ± 2.3 | -107.7 ± 15.4 | -19.2 ± 3.3 | 1609 ± 28 |
| V483A | 38 (0.04% | -94.8 ± 2.6 | -54.2 ± 2.3 | -98.8 ± 13.4 | -20.8 ± 2.3 | 1598 ± 52 |
| E484K | 10 (0.01%) | -94.6 ± 3 | -56.3 ± 2.8 | -90.4 ± 11.4 | -20.3 ± 3.5 | 1633 ± 43 |
| E484Q | 18 (0.02%) | -97 ± 2.2 | -56.6 ± 1.5 | -93.1 ± 16.9 | -21.8 ± 2.6 | 1600 ± 28 |
| G485R | 21 (0.02%) | -97.1 ± 1.3 | -55.6 ± 2.5 | -103.7 ± 8.9 | -20.8 ± 1.8 | 1620 ± 64 |
| F486L | 32 (0.04%) | -82.1 ± 2.2 | -51.2 ± 2.8 | -98.4 ± 14 | -11.2 ± 2.2 | 1560 ± 62 |
| S494P | 30 (0.03%) | -94.5 ± 2 | -55.3 ± 2.1 | -88 ± 16.7 | -21.6 ± 1.2 | 1628 ± 33 |
| Q498H | 17 (0.02%) | -102 ± 1.6 | -56.3 ± 2.8 | -110.1 ± 11 | -23.7 ± 3.2 | 1637 ± 22 |
| N501Y | 39 (0.04%) | -101.4 ± 4.3 | -59.2 ± 3.4 | -91.1 ± 12.4 | -24 ± 3.7 | 1648 ± 60 |
